# Efficacy of Mobile Health for Self-management of Cardiometabolic Risk Factors: A Theory-Guided Systematic Review

**DOI:** 10.1097/JCN.0000000000000659

**Published:** 2020-02-10

**Authors:** Sabianca Delva, Kyra J. Waligora Mendez, Mia Cajita, Binu Koirala, Rongzi Shan, Shannon Wongvibulsin, Valerie Vilarino, Danielle R. Gilmore, Hae-Ra Han

**Affiliations:** **Sabianca Delva, BSN, RN** PhD Candidate, School of Nursing, The Johns Hopkins University, Baltimore, Maryland.; **Kyra J. Waligora Mendez, BSN, RN** PhD Candidate, School of Nursing, The Johns Hopkins University, Baltimore, Maryland.; **Mia Cajita, BSN, RN** Postdoctoral Fellow, Department of Health and Community Systems, School of Nursing, University of Pittsburgh, Pennsylvania.; **Binu Koirala, BSN, RN** PhD Candidate, School of Nursing, The Johns Hopkins University; and Center for Innovative Care in Aging, The Johns Hopkins School of Nursing, Baltimore, Maryland.; **Rongzi Shan** Research Fellow, MD Candidate, Johns Hopkins University School of Medicine, Baltimore, Maryland; and David Geffen School of Medicine at University of California Los Angeles.; **Shannon Wongvibulsin, BS** MD-PhD Candidate, Johns Hopkins University School of Medicine, Baltimore, Maryland.; **Valerie Vilarino** BS Candidate, Johns Hopkins Krieger School of Arts and Sciences, Baltimore, Maryland.; **Danielle R. Gilmore, MPP** PhD Student, Tratchenberg School of Public Policy & Administration, George Washington University, Washington, DC.; **Hae-Ra Han, PhD, RN, FAAN** Professor School of Nursing, The Johns Hopkins University; and Center for Cardiovascular and Chronic Care and Center for Community Innovation and Scholarship, The Johns Hopkins School of Nursing, Baltimore, Maryland.

**Keywords:** cardiometabolic risk factors, cardiovascular disease, metabolic syndrome, mobile health (mHealth), telemedicine

## Abstract

**Background:**

Although mobile health (mHealth) technologies are burgeoning in the research arena, there is a lack of mHealth interventions focused on improving self-management of individuals with cardiometabolic risk factors (CMRFs).

**Objective:**

The purpose of this article was to critically and systematically review the efficacy of mHealth interventions for self-management of CMRF while evaluating quality, limitations, and issues with disparities using the technology acceptance model as a guiding framework.

**Methods:**

PubMed, CINAHL, EMBASE, and Lilacs were searched to identify research articles published between January 2008 and November 2018. Articles were included if they were published in English, included adults, were conducted in the United States, and used mHealth to promote self-care or self-management of CMRFs. A total of 28 articles were included in this review.

**Results:**

Studies incorporating mHealth have been linked to positive outcomes in self-management of diabetes, physical activity, diet, and weight loss. Most mHealth interventions included modalities such as text messaging, mobile applications, and wearable technologies. There was a lack of studies that are (1) in resource-poor settings, (2) theoretically driven, (3) community-engaged research, (4) measuring digital/health literacy, (5) measuring and evaluating engagement, (6) measuring outcomes related to disease self-management, and (7) focused on vulnerable populations, especially immigrants.

**Conclusion:**

There is still a lack of mHealth interventions created specifically for immigrant populations, especially within the Latino community—the largest growing minority group in the United States. In an effort to meet this challenge, more culturally tailored mHealth interventions are needed.

Cardiovascular disease places a significant public health burden on the US healthcare system.^1^ Cardiometabolic risk factors (CMRFs) are a cluster of risk factors, including obesity, high fasting blood sugar, hypertension, and high triglycerides that increase the risk of cardiovascular disease and diabetes.^1^ Adjusted annual healthcare expenditures are approximately double for those with 3 or 4 CMRFs compared with those with 0 or 1 CMRF.^2^ Moreover, racial disparities exist within cardiovascular care where blacks and Hispanics have lower cardiovascular disease treatment rates than non-Hispanic whites.^3,4^ Mobile health (mHealth) technologies are innovative healthcare delivery mechanisms that may improve self-management of CMRFs.

Mobile phone ownership and Internet access have drastically increased^4^; 95% of the US population owns mobile phones.^5^ When adopted, mHealth interventions are effective in improving treatment adherence and health outcomes, especially CMRFs.^6,7^ Common mHealth modalities include text messaging–facilitated patient-provider communication, smartphone mobile applications, wearable technologies, and medical peripheral devices to monitor and access health-related information. Interventions using cell phones, smartphone apps, and text messaging resulted in improved self-care, adherence to treatment,^8^ improved self-management,^9^ and healthcare savings.^9^ Despite the promising potential of mHealth to improve self-management of CMRFs, its use in clinical and real-world settings is unrealized—partly because of the lack of systematic evidence of its efficacy.

As an immediate first step, it is important to examine and synthesize research regarding self-management of CMRFs using mHealth. In this review, we (1) evaluated the efficacy of existing mHealth interventions targeting self-management of CMRFs, (2) identified factors associated with adoption of successful mHealth interventions in CMRF management, and (3) reviewed disparities in mHealth research for self-management of CMRFs. Specifically, we used the technology acceptance model as a framework to systematically identify social, structural, and systematic barriers and facilitators to mHealth adoption.

## Theoretical Framework

**FIGURE 1:**
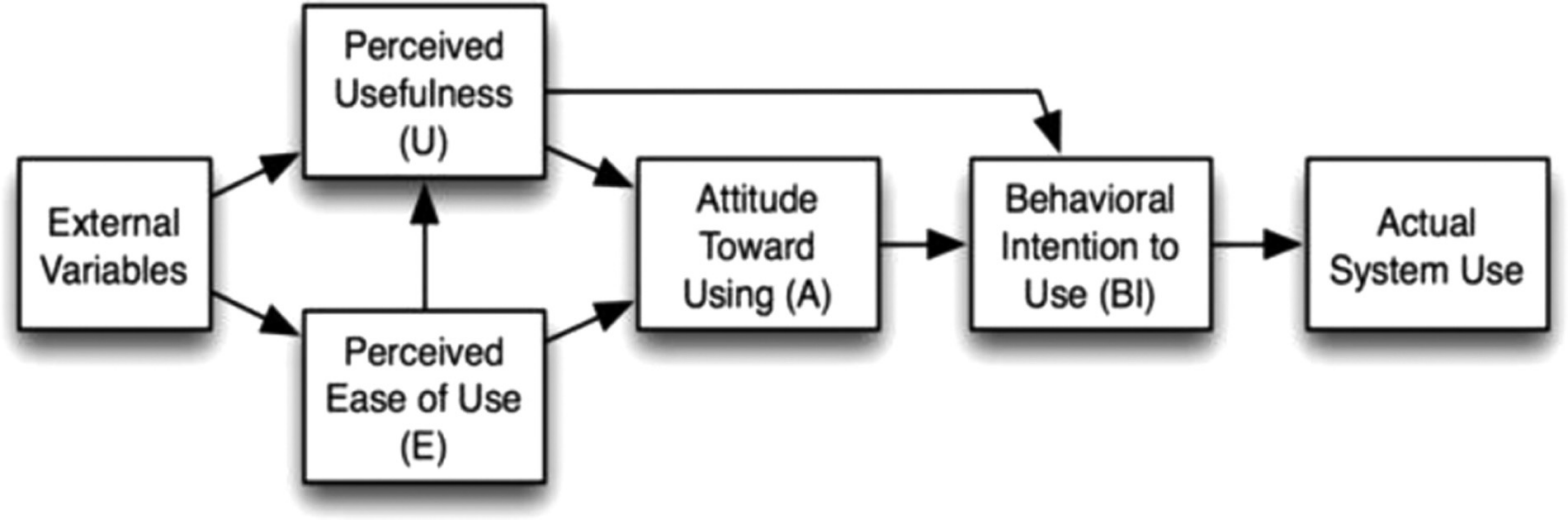
Technology acceptance model.^11^

Previously published systematic reviews and meta-analyses have demonstrated the benefit of using a framework for integration of data to assess relationships between constructs and variables.^10^ We used the technology acceptance model^11^ to guide this review's exploration of how perceptions, attitudes, and intentions influence mHealth adoption among people with CMRFs (see Figure 1). The model uses the following constructs to identify predictive factors in participants' adoption of mHealth: perceived usefulness, the “subjective probability that using a specific application system will increase job performance,” perceived ease of use, “the degree to which the [...] user expects the target system to be free of effort,”^11^^(p985)^ attitude toward using the system, behavioral intention to use, and actual adoption.

## Methods

### Search Methodology

This systematic review is reported according to the Preferred Reporting Items for Systematic Reviews and Meta-Analyses guidelines.^12^ A comprehensive search was carried out in the Cumulative Index to Nursing and Allied Health Literature, PubMed, EMBASE, and Lilacs databases for articles published between January 2008 and October 2018 to identify literature on mHealth interventions to improve self-management among populations with CMRFs. We restricted our scope to studies conducted in the United States to capture healthcare disparities among groups such as racial and ethnic minorities or those who are immigrants living in the United States.^3,4^ In consultation with a medical librarian, the following terms were included in the PubMed search, with similar terms used in the other databases: “telehealth,” “Telemedicine,” “mobile health,” “ehealth,” “mhealth,” “Metabolic Syndrome X,” “Cardiovascular Disease(s),” “cardiac risk factor,” “risk factors.”

**FIGURE 2:**
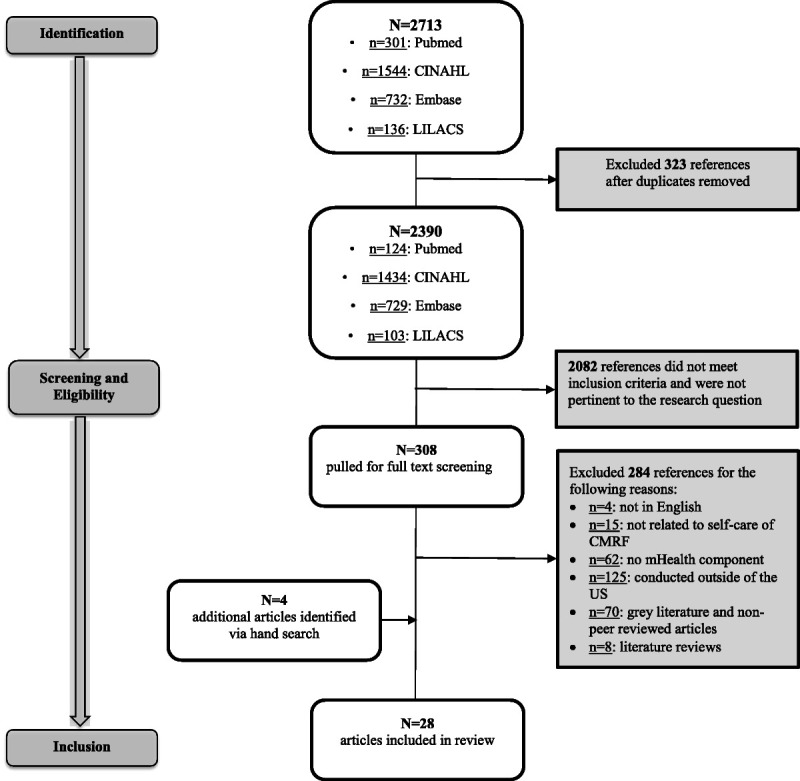
Diagram of article selection process with an explanation of search strategy up to October 2018. Four additional articles were identified via hand search in November 2018. Twenty-eight articles were included in the literature review.

Studies were included if they (*a*) were published in English, (*b*) used an mHealth intervention, (*c*) addressed self-care of any type of CMRF, (*d*) sampled adults, and (*e*) were conducted in the United States. Articles were excluded if they (*a*) were abstracts, (*b*) were nonresearch articles (eg, review articles, editorial, protocol papers), and (*c*) investigated mHealth but did not relate to self-care of CMRFs (eg, clinician-delivered intervention, health coaching via telephone) (see Figure 2).

### Interrater Agreement

Two authors independently reviewed titles, abstracts, and full texts to determine eligibility. For title and abstract screening, the levels of agreement were moderate, ranging from 47.3% to 55.8%.^4^ For full-text screening, the indices of agreement were all considered to be good, ranging from 60% to 69.2%. A third rater adjudicated any discrepancy or conflicts between reviewers. Two reviewers independently assessed risk of bias for each study. An 85% agreement rate between reviewers was reached. Discordance was resolved by a vote from a third reviewer.

## Results

### Screening and Selection of Articles

Figure 1 shows the article screening and selection process. The electronic search returned 2713 articles, of which 323 were duplicates. Of the remaining 2390 articles, 2082 did not meet the inclusion criteria. The remaining 308 articles were pulled for full-text screening, of which 284 were excluded for reasons indicated in Figure 2. Four new articles were added via hand search for full-text review in November 2018. A total of 28 articles were included in this review.

### Characteristics of Included Studies

The designs of the 28 mHealth-related studies were the following (Table 1):

**TABLE 1:** Summary of Selected Studies, Quality Rating, Design, Follow-up, and Study Characteristics and Purpose

Author(s), Year	Setting	Study Design/Duration	Study Characteristics and Demographic Information					Purpose	Level of Evidence (I, II, III, or IV)
			N	Mean Age, y	Sex, n, %	Ethnicity, n, %	Disease		Quality Rating: (Low, Good, or High)
Alshurafa et al,^13^ 2017	One church and the surrounding community in an urban LA area	RCT 3- and 6-mo follow-up visits	37	Mean age not reported Range, 25–45	Female (100)	Black (100)	Risk factors for CVD	To describe an enhanced Remote Health Monitoring system, Wanda-CVD, that provides wireless coaching.	Level II Medium
Arora et al,^14^ 2014	ED at LA County Hospital of the University of Southern California	RCT 6 mo	128	50.7	Male (36) Female (64)	Hispanic/Latino (87) Black (9) White (2) Asian/Pacific Islander (2)	DM	To evaluate a daily text message intervention, TExT-MED, for resource-poor ED patients.	Level I High
Austin et al,^15^ 2012	Private not-for-profit hospital in Charleston, South Carolina	RCT 6 mo	60	64.5	Male (38.3) Female (61.7)	White (51.7) AA (46.7) Hispanics (1.7)	CHF	To determine whether an interactive voice response system with daily messages would be well accepted by patients and reduce readmissions.	Level I High
Brewer et al,^16^ 2018	Five AA churches in southeast Minnesota	Quasi experimental 6 mo	50	49.6	Male (30) Female (70)	AA (100)	CVDs	To deliver health education and motivational support to users to improve CV health via *FAITH!* app.	Level II High
Dang et al,^17^ 2010	Telehealth clinic at the Veterans Affairs Medical Center in Miami, FL	Prospective quasi-experimental cohort (no control group) comparing baseline Framingham risk score (FRS) to FRS at 2 y	41	68.7	Male (93) Female (7)	White (41.5) AA (26.8) Hispanics (31.7)	T2DM, HBP, high cholesterol	To determine the impact of telehealth care coordination (T-Care) program on CHD risk in older veterans.	Level II High
Duscha et al,^18^ 2018	Cardiac rehabilitation (CR) center at Duke University Medical Center, Durham, NC	RCT; 3:1 randomization to mHealth vs UC 12 wk	25	59.9 in mHealth arm, 66.5 in UC	Male (81.2) in mHealth arm Male (66.7) in UC	Black (31.2) Non-Hispanic white (68.8)	CVDs	To determine the effects of an mHealth-based program using smartphones, physical activity (PA) trackers and health coaching for graduates of a center-based CR program on PA and peak oxygen uptake	Level I High
Ferguson et al,^19^ 2010	CHF clinic, University of Rochester Medical Center in Rochester, NY	Cross-sectional; focus group (FG), survey	9 FG/63 survey	Range, 35–82 in FG; 54.8 in survey	Not stated	CHF	To describe the prototyping and design process of a conversational assistant to help monitor subjective and objective observations.	Level III Low
Forjuoh et al,^20^ 2014	Seven regional clinics of a university-affiliated health maintenance organization (HMO) practice in Central TX	4-arm nonblinded RCT 12- and 24-mo follow-up visits	376	57.6	Male (44.9) Female (55.1)	Hispanic (20.2) Non-Hispanic black (16.2) Non-Hispanic white (60.1) Other (3.5)	T2DM	To compare the effectiveness of classroom-based versus mHealth-delivered DM education on HbA_1c_ in an ethnically diverse HMO.	Level I High
Fortmann et al,^21^ 2017	Clinics within a network of federally qualified health centers in San Diego and Riverside, CA	2-arm nonblinded RCT 3- and 6-mo follow-up visits	126	47.8 in Dulce Digital (DD), 49.1 in UC	Male (13.5) and female (36.5) in the DD arm Male (11.9) and female (38.1) in UC	Mexican (91), US-born (5), other (4)	T2DM	To evaluate the effect of DD, an SMS-delivered diabetes education intervention versus UC.	Level I High
Frias et al,^22^ 2017	13 outpatient primary care facilities across CA and CO	3-arm, cluster RCT 12 wk	109	57.8 in combined digital medicine offering (DMO), 61.6 in UC	Female, n = 45, in combined DMO arm; female, n = 10, in UC	In combined DMO: AA (12.8), Hispanics (33.9), white (48.6), Asian (11.9) In UC: AA (2.75), Hispanics (12.8), white (17.4), Asian (1.83)	HBP and T2DM	To assess the impact on clinic-measured blood pressure (BP) and glycated hemoglobin (HbA_1c_) using a DMO that measures medication ingestion adherence, PA, and rest using digital medicines (with ingestible sensor), wearable sensor patches, and a mobile device application.	Level I High
Fukuoka et al,^23^ 2015	Primary care clinics in San Francisco and Berkeley, CA	RCT 5 mo	61	55.2	Female (77)	Racial/ethnic minorities (48)	T2DM and OW	To examine the feasibility and efficacy of a DM prevention intervention combined with a mobile app (mDPP) and pedometer in English-speaking OW adults at risk for T2DM.	Level I High
Gilmore et al,^24^ 2017	Women, Infants, and Children (WIC) services clinics in Baton Rouge, LA.	Prospective, parallel-arm, RCT 16 wk	35	26	Female (100)	In E-Moms: black, n = 14; white, n = 2; Asian, n = 0 In WIC Moms: black, n = 12; white, n = 6; Asian, n = 1	OW and OB	To test the efficacy of a smartphone-based intervention, “E-Moms” versus UC or “WIC Moms,” to promote postpartum weight loss.	Level I High
Glasgow et al,^25^ 2011	Primary care clinics within Kaiser Permanente, CO	Three-arm RCT 4 mo	270	57.8	Female (48.1)	American Indian/Alaska Native (4.2), Asian (1.5), black (18.1), white (67.4), other (8.9) Latino ethnicity (22.3)	DM, OW, CVD risk factor	To report on (1) the overall rate of use of the My Path/Mi Camino diabetes self-management website, (2) the frequency of engagement with website components, (3) participant characteristics and their associations with greater engagement with the website, and (4) the relations between measures of engagement and 4-mo outcomes.	Level I High
Graziano,^26^ 2008	2 clinics (primary care clinic and endocrinology clinic) at an urban medical center in the Midwest.	RCT 3 mo	120	60.1 in the telephone group (TG), 63 in CG	In TG: male, n = 33; female, n = 28 In CG: male, n = 33; female, n = 25	In TG: white, n = 43; nonwhite, n = 18 In CG: white, n = 49; nonwhite, n = 9	T2DM	To evaluate the effect of an easily implemented, automated telephone intervention on glycemic control in patients with type 2 DM.	Level I High
Han et al,^27^ 2018	Community locations in an urban inner city	Quasi-experimental; feasibility 16 wk	11	54.7	Female (63.6)	Hispanic, 11 (100)	HBP	To develop a health literacy–focused intervention for Latinos—PLAN 4 Success-HBP	Level II High
Kim et al,^28^ 2016	Scripps Health clinics	RCT; 2-group, pre-post trial 6 mo	95	57.6	Female (68)	Caucasian (80), AA (6), Hispanic (5), Asian (5)	HBP, DM, cardiac dysrhythmia	To determine use of wireless self-monitoring program on patient activation and health behaviors, medication adherence, and control of BP vs control group	Level I High
Martin et al,^29^ 2015	Outpatients at an academic CVD prevention center in Baltimore, MD	Sequential randomization 5 wk	48	58	Female (46)	White (79), non-white (21)	CVD	To evaluate an mHealth intervention, mActive, that provides individual encouragement foster feedback loops and increases PA.	Level I High
McGillicuddy et al,^30^ 2015	Medical University of Charleston, SC.	Retrospective RCT 12 mo	18	42.44 in the IG, 57.89 in the CG	Female, n = 13; male, n = 5	Black, n = 14; white, n = 3; Hispanic, n = 1	HBP in kidney transplant recipients	To evaluate preliminary indications of sustainability of improved BP in kidney transplant recipients 12 mo after completion of a 3-mo RCT of mHealth pilot program.	Level I High
Morawski et al,^31^ 2017	Large medical center in Boston, MA	Prospective RCT 12 wk	411	52	Female (60)	Black (25)	HBP	To evaluate impact of mHealth application (Medisafe) on BP and medication adherence. Patients randomized in 1:1 fashion to UC vs Medisafe mHealth platform.	Level I High
Naslund et al,^32^ 2016	Urban community mental health center in southern NH	Exploratory study, single arm (pre/post) 6 mo	34	50.2	Female (61.8)	Non-Hispanic white, n = 34 (100)	OW and OB	To examine whether daily step count measured using a wearable accelerometer is associated with weight loss and improved fitness	Level III High
Park et al,^33^ 2014	Nonprofit, community hospital in northern CA	Prospective, 3-arm RCT 30 d	90	59.2	Male (76)	Nonwhite (22)	Chronic heart disease	To test the efficacy of an mHealth intervention using text messaging to improve medication adherence	Level I High
Sepah et al,^34^ 2015	Internet-based diabetes prevention program	Quasi-experimental, prospective, single-arm, preintervention and postintervention study 2 y	220	43.6	Male, n = 38	White, n = 108 (50.2); black, n = 63 (29.3); Hispanic, n = 23 (10.7); other, n = 21 (9.8)	Pre-DM; OW and OB	To investigate the long-term outcomes and sustainability of an Internet-based DM prevention program	Level II High
Shane-McWhorter et al,^35^ 2014	4 rural and 2 urban primary care clinics, and 1 urban stroke center, UT	Quasi-experimental, prospective, observational pre-and-post study 6 mo	109	50.6	Female (64)	Primary language: Spanish, n = 72 (66.1); English, n = 37 (33.9)	DM, HBP	To use telemonitoring devices to expand and improve chronic disease management of patients with DM and/or HBP	Level II High
Sieverdes et al,^36^ 2015	Dialysis Clinic, Inc, facilities in Charleston, SC	Qualitative interviews	22	46	Female (45)	AA (82)	Kidney disease	To explore barriers and perceptions of PA behaviors and gauge interest in using mHealth in a PA wellness program for patients waiting for kidney transplant.	Level III High
Sieverdes et al,^37^ 2017	Family medicine practice and college campus in a southeastern coastal city in the United States	Mixed methods A qualitative approach consisting of 4 FGs and a battery of questionnaires were used.	34	43.1	Female (58.8)	White, n = 18 (52.9); AA, n = 15 (44.1); other, n = 1 (2.9)	Adults with preessential HBP (preEH)	To identify whether a culturally tailored approach is needed in the design and preferences between groups of preEH African American and white adults toward using a smartphone BAM app, the Tension Tamer app.	Level III High
Skolarus et al,^38^ 2018	Churches in Flint, Michigan	Randomized, pilot intervention trial	94	58	Female, n = 90 (79)	AA, n = 92 (97)	HBP	To assess the feasibility of the Reach Out processes, a faith-collaborative, mobile health, randomized, pilot intervention trial of 4 mobile health components to reduce high BP compared with usual care.	Level I High
Staffileno et al,^39^ 2018	University medical center and community clinics	Randomized, pre-post design 12 wk	26	In DASH arm, 35.3; in PA arm, 35.1	Female (100)	AA (100)	PreEH	To evaluate a healthy lifestyle intervention delivered using an eHealth platform, targeting young AA women at risk for developing HBP to promote a healthy lifestyle through increased PA and improved nutrition.	Level I High
Svetkey et al,^40^ 2015	Locations in 3 specific counties in North Carolina (Durham, Orange, and Wake)	RCT 24 mo	365	Mean not stated; range, 18–35	Male (30)	AA (30), Latinos (6)	OB	To compare 2 interventions ([1] cell phone intervention, [2] personal coaching intervention) for weight loss to a usual-care control group.	Level I High

Abbreviations: AA, African American; CG, control group; CHD, coronary heart disease; CHF, coronary heart failure; CV, cardiovascular; CVD, cardiovascular disease; DASH, Dietary Approaches to Stop Hypertension; DM, diabetes mellitus; ED, emergency department; HbA_1c_, hemoglobin A_1c_; HBP, high blood pressure; IG, intervention group; OB, obesity; OW, overweight; PA, physical activity; RCT, randomized controlled trial; SMS, text messages; T2DM, type 2 diabetes mellitus; UC, usual care.

○ Quantitative (n = 25): randomized controlled trials (n = 19)^13–15,18,20–26,28–31,33,38–40^ and quasi-experimental studies (n = 6)^16,17,27,32,34,35^
○ Qualitative interviews (n = 1)^36^
○ Mixed methods (n = 2)^19,37^

The studies investigated mHealth interventions targeting various CMRFs, including high blood pressure (HBP), high cholesterol, overweight and obesity, and diabetes, as well as cardiovascular disease, congestive heart failure, and kidney disease. The follow-up period for the randomized controlled trials and quasi-experimental studies ranged from 30 days to 24 months. Follow-up periods averaged within 1-, 3-, and 6-month increments, with only 1 study having shorter weekly posttest windows.^29^

Six studies were conducted in urban settings^13,26,27,29,32,35^; and one, in a rural setting.^35^ Participants were recruited from large academic medical centers (n = 8),^14,15,17,30,31,33,39,40^ primary care and outpatient clinics (n = 13),^19–26,28,35–37,39^ cardiac rehabilitation (n = 1),^18^ churches (n = 3),^13,16,38^ and an online community (n = 1).^34^ Clinical conditions contributing to cardiovascular diseases included general cardiac risk factors (n = 5),^13,16–18,25,29^ hypertension (n = 9),^22,27,28,30,31,35,37–39^ coronary heart disease (n = 2),^28,33^ congestive heart failure (n = 2),^19,20^ diabetes (n = 11),^14,17,20–23,25,26,28,34,35^ kidney disease (n = 1),^36^ and obesity/overweight (n = 6).^23–25,32,34,40^ All of the studies included participants 18 years and older, with an age range of 26 to 65 years. Sample sizes ranged from 11 to 411 with a mean of 109. Most studies had representation from both men and women, whereas 3 studies targeted a female-only sampling frame.^13,24,39^ Given this article's focus on health disparities, we also report how many studies recruited from underserved populations: federally qualified health center (n = 1),^30^ Women, Infants, and Children clinic (n = 1),^24^ uninsured (n = 1),^21^ safety-net emergency department (n = 1),^14^ veterans (n = 1),^17^ mentally ill (n = 2),^22,32^ and low-income individuals (n = 3).^14,21,22^ In terms of ethnicity, 24 studies had a heterogeneous sample of ethnic minorities with the exception of a few that sampled only white (n = 1),^32^ black (n = 3),^13,16,39^ or Hispanic/Latino (n = 1) participants.^27^ Overall, 8 studies culturally tailored their intervention to vulnerable groups.^13,16,20,25,27,37–39^

### Quality Appraisal

The Johns Hopkins Research Evidence Appraisal Tool was used to assess the quality of the included studies (Table 1).^41^ Two team members (S.D. and K.W.) independently reviewed and scored the studies identified from the literature search. The quality ratings were then combined, and any studies that lacked a clear majority agreement were resolved by discussion. Remaining disagreements were adjudicated by a third author (H.H.). Among the selected studies, those without interventions or with a qualitative component were ranked level III.^19,32,36,37^ Articles ranked level II were quasi-experimental studies, where there was a lack of control group and/or no randomization.^13,16,17,27,34,35^ Nonetheless, despite some minor limitations, the quasi-experimental studies were strong in design and statistical analysis, because they controlled for confounding variables and systematic bias. Studies with sample sizes that were sufficient for their study design, were conducted with robust methods, and had strong analyses yielding statistical significance were given high-quality ratings and ranked level I.^14,15,18,20–26,28–31,33,38–40^

Raters standardized the score to range from 0 to 10 because not all questions were applicable. The average rating of quality scores for the 19 randomized controlled trials was 8.8 of 10 (range, 7–10). Twenty-six of 28 studies were rated high-quality (6.68 or higher), 1 study was in the medium-quality category (scores of 3.34–6.67),^13^ and one was rated low (0–3.33).^19^ Seven quasi-experimental studies had an average quality rating of 8.5 (range, 7.5–10; maximum possible score, 10), and they all met the criterion of being high-quality (7 or greater).^13,16,17,27,32,34,35^

### Report on Risk of Bias

**FIGURE 3:**
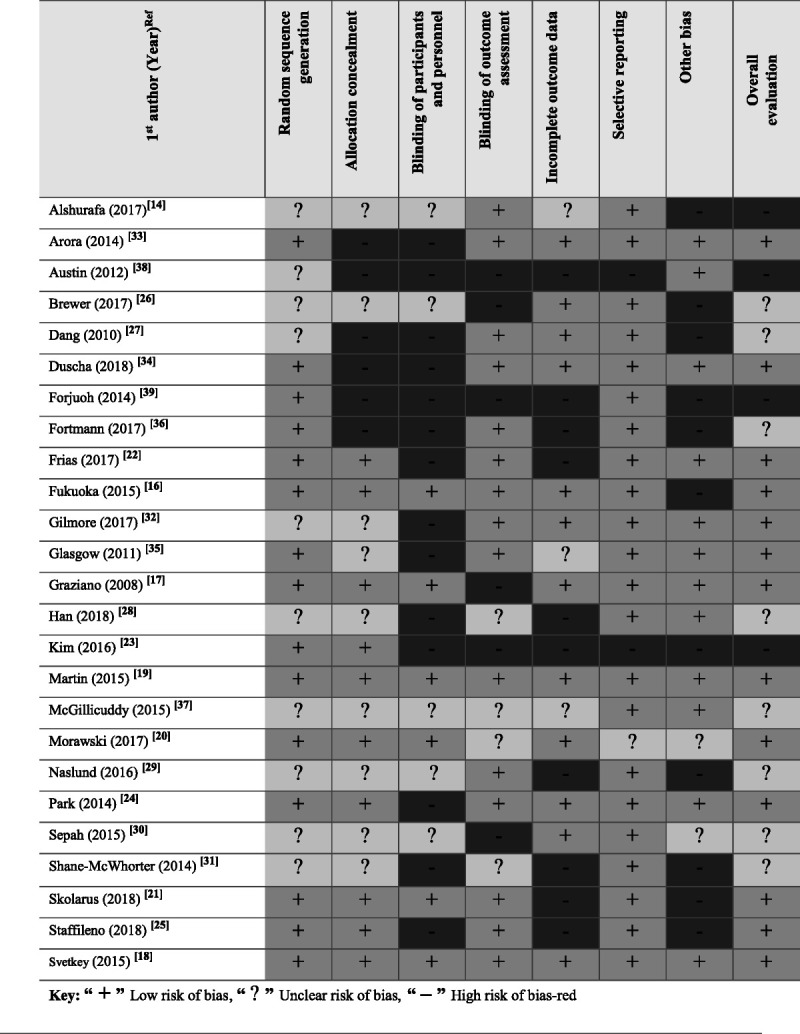
Risk of bias for selected studies.

**FIGURE 4:**
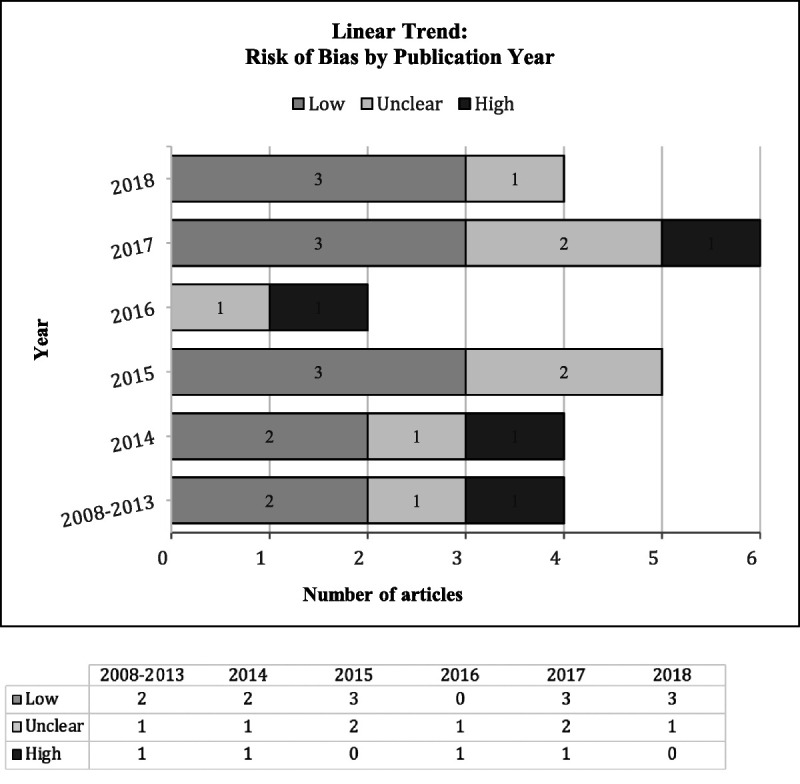
Risk of bias for published mHealth interventions has decreased for a period of 10 years.

The Cochrane Collaboration's Risk of Bias tool for randomized controlled trials^42^ was used to evaluate risk of bias across the following domains: allocation concealment, blinding (participants, outcome assessors, investigators) for subjective outcomes, and justification for incomplete outcome data (Figure 3). Of the 25 randomized controlled trials and quasi-experimental studies, 12 studies had a low risk of bias,^14,18,22–26,29,31,33,38–40^ 9 had an unclear risk of bias,^16,17,21,27,30,32,34,35^ and 4 had a high risk of bias.^13,15,20,28^ A linear trend was performed with descriptive statistics to assess validity of the randomized controlled trials and quasi-experimental studies over time.^28,43^ Data on risk of bias were merged for all years below 2014 and summarized by year and type of bias. We calculated bias percentage within year and reported the results in frequency and proportions (Figure 4). We found risk of bias for mHealth studies decreased over a decade (2008–2018), suggesting that researchers are becoming more diligent about randomization, blinding, and allocation procedures in this burgeoning research arena.

### Mobile Health Interventions: Modalities and Features

#### Mobile Health Modalities

We report on whether the study designs were theory based, the types of mHealth modalities used, and study outcomes in Table 2. Ten studies were driven by health promotion theories or a theoretical framework.^14,16,24–26,29,30,38–40^ Mobile health modalities included websites (n = 4),^21,28,34,39^ text messages (n = 11),^13,14,18,21,25–27,29,30,33,38^ smartphone apps (n = 12),^13,16,18,20,22–24,28,30–32,40^ voice technology (n = 4),^15,17,26,35^ and digital medication tracking system (n = 3).^22,30,33^ Given the focus on promoting self-care, participants were encouraged to use different forms of wearable technologies (n = 8),^13,18,22,24,29,32,39,40^ such as sensor-enabled devices, wireless or Bluetooth-enabled scales, and smart fitness trackers.

**TABLE 2:** Mobile Health Modalities, Self-care Outcomes, and Use of Theories to Guide Study Components in Intervention-Based Studies

First Author	Theory Based	mHealth Intervention(s)	Self-care Measure(s)	Outcomes/Results
Alshurafa^a^^13^		Smartphone-based remote health monitoring system, SMS, tracking	CVD risk, healthy eating	Factors such as the variation in first-month intervention response to the consumption of nuts, beans, and seeds in the diet help predict patient RHM protocol outcome success in a group of young black women ages 25–45 y.
Arora^14^	**Social cognitive theory**	Daily unidirectional text message	HbA_1c_ and MA	Median HbA_1c_ decreased by 1.05% in the TExT-MED group compared with 0.60% in the CG (SD, 0.45; 95% confidence interval [CI], –0.27 to 1.17). MA was improved from 4.5 to 5.4 in the TExT-MED group compared with a net decrease of −0.1 in the controls (SD, 1.1; 95% CI, 0.1–2.1).
Austin^15^		Daily voice messages	CHF readmissions	Readmission rate of 10% compared with the Roper baseline CHF readmission rate of 21% (*P* = .047).
Brewer^16^	**Behavioral theory**	Faith app	HL	Participants had high EHL (84.8% [39/46] with eHEALS score ≥ 26) with no differences by sex.
Dang^17^		In-home messaging device	CHD risk via FRS	Significant reductions in FRS (23.4 ± 13.5 to 18.2 ± 10.4, *P* = .007), systolic BP (140 ± 22.7 to 128.2 ± 18.5 mm Hg, *P* = .05), and diastolic BP (74 ± 13.8 to 68.7 ± 13.9 mm Hg, *P* = .07), but not in LDL cholesterol (100.2 ± 30.1 to 91.2 ± 26.6 mg/dL, *P* = .7).
Duscha^18^		Coaching via the Vida mobile app, Fitbit, SMS messages	PA and peak VO_2_	Change in peak VO_2_ after 12 wk was different between mHealth (4.7% ± 13.8%) and UC (−8.5% ± 11.5%, *P* < .05). Low and high PA decreased in UC (*P* < .05). Nonsignificant increase of moderate-high activity in the mHealth IG.
Forjuoh^20^		PDA-delivered diabetes self-care software	GC	HbA_1c_ reductions at 12 mo for the groups averaged 1.1%, 0.7%, 1.1%, and 0.7%, respectively, but did not differ significantly from baseline (*P* = .771). No marked reductions in HbA_1c_ for minority persons but rather a reduction for all racial/ethnic groups.
Fortmann^21^		Daily SMS messages	GC	The Dulce Digital group had a significantly greater reduction in HbA_1c_ over time compared with UC (*P* = .03). The number of blood glucose values texted back by participants predicted month 6 HbA_1c_ (*P* < .05).
Frias^22^		Digital medicines, wearable sensor patch, and mobile app	HBP and GC	At week 4: - Combined DMO had a mean change in SBP of −21.8 mm Hg compared with −12.7 mm Hg for UC. -More DMO participants achieved their BP goal (81%) compared with UC (33.3%). -DMO participants had a greater reduction in DBP compared with UC, but the results were not significant. At week 12: -98% of DMO participants achieved their BP goal compared with 51.7% of UC participants. -At week 12, DMO had a nonsignificant difference in HbA_1c_ reduction compared with UC. Both weeks 4 and 12 DMOs with a baseline HbA_1c_ of 8% or more showed larger HbA_1c_ decreases than UC.
Fukuoka^23^		Mobile phone app; pedometer	WL, WC, PA, HBP, healthy eating, and cholesterol	The IG lost an average of 6.2 kg between baseline and 5-mo follow-up compared with the CG's gain of 0.3 kg. The IG's steps per day increased by 2551 compared with the CG's decrease of 734 steps per day. The IG had greater reductions in hip circumference, BP, and intake of saturated fat and sugar-sweetened beverages. The intervention had no significant effect on fasting lipid or glucose levels.
Gilmore^24^	**Self-efficacy**	Smartphone-based application, Fitbit	Postpartum WL, WC, HC, BP	No difference in WL and WC between the IG and CG; however, those who had greater than 70% adherence to the intervention had significant WL (−3.6 ± 1.6 vs 1.8 ± 0.9 kg; *P* = .005) and change in HC (−5.0 cm, *P* = .006). No change from baseline SBP (*P* = .96) and DBP (*P* = .54) between the CG and IG.
Glasgow^25^	**Social-ecological theory**	Website support; SMS messages, phone calls	PA, healthy eating, MA, HL	Website use was most consistently related to the dietary measures. There was also a significant relation between self-monitoring and improvement in physical activity but not with medication adherence.
Graziano^26^	**Health-behavior theory**	Prerecorded daily voice message and SMS	GC	No significant differences between the telephone group and the CG on mean change HbA_1c_ level (*P* = .84), suggesting no treatment effect.
Han^27^		Monthly phone counseling; optional text messaging	HL, HBP, and MA	Mean changes in SBP and DBP were decreases of 24.1 and 11.3 mm Hg, respectively; 91% participants achieved BP control (<140/90 mm Hg). For health literacy, the effect sizes ranged from 0.1 to 1.7 in absolute value. The number of participants taking HBP medication increased from baseline to 16 wk (from n = 3 to n = 5).
Kim^28^		Web-based disease management program; mobile app for monitoring and education	MA and HBP	Improvements in patient activation were associated with improvements in BP control (β = 0.04, *P* = .02). This relationship was further strengthened in reducing cigarettes (β = −0.60, *P* < .001), alcohol drinking (β = −0.26, *P* = .01), and SBP (β = −0.27, *P* = .02) and DBP (β = −0.34, *P* = .007). No differences were observed with respect to MA.
Martin^29^	**Behavior change theory**	mHealth intervention with tracking; texting components	PA	The phase I change in PA was not significantly higher in unblinded participants versus blinded controls by 1024 daily steps (95% CI, 580–2628; *P* = .21). In phase II, participants receiving texts increased their daily steps over those not receiving texts by 2534 (95% CI, 1318–3750; *P* < .001) and over blinded controls by 3376 (95% CI, 1951–4801; *P* < .001).
McGillicuddy^30^	**Self-determination and behavior change theories**	Smartphone application, electronic medication tray, SMS messages	HBP	The IG group exhibited lower SBP at the 12-mo follow-up visit (*P* = .01) compared with the CG. At the 12-mo follow-up, success in establishing and sustaining control of SBP (<131 mm Hg) was greater in the IG (50%) than in the CG (11%).
Morawski^31^		mHealth application (Medisafe)	BP and MA	After 12 wk, the mean (SD) score on the MMAS improved by 0.4 (1.5) among the IG and remained unchanged among the CG (between-group difference, 0.4; 95% CI, 0.1–0.7; *P* = .01). After 12 wk, the mean (SD) SBP decreased by 10.6 (16.0) mm Hg among the IG and 10.1 (15.4) mm Hg among the CG (between-group difference, −0.5; 95% CI, −3.7 to 2.7; *P* = .78).
Naslund^32^		Wearable accelerometer and Fitbit application	WL, PA, and fitness	Every 1000-step increase in participants' daily average step count was associated with a decrease of 1.78 lb (*P* = .0314). An increase of 1000 steps corresponded to an increase of 18.79 ft on the 6-Minute Walk Test; however, it was not significant (*P* = .176).
Park^33^		Daily SMS messages, medication monitoring via electronic pills	MA	-The “SMS reminders + SMS education” group had a higher percentage of prescribed doses taken (*P* = .02) and percentage of doses taken on schedule (*P* = .01) for antiplatelet medications. -The “SMS education alone” group had a higher percentage of number of doses taken compared with the “no SMS” group (*P* = .01). No significant differences were found among the 3 groups over time for self-reported medication adherence. -Comparing the “SMS reminders + SMS education” and “no SMS” groups, the effect size of the intervention was medium to large (Cohen *d* = 0.69).
Sepah^34^		Internet-based education	GC, WL	Weight change: -Program starters (n = 187, completed at least 4 core lessons) achieved a mean weight loss of 4.2% from baseline to year 2. -Program completers (n = 155, completed at least 9 core lessons) achieved a mean weight loss of 4.3% from baseline to year 2. HbA_1C_: -Program starters (n = 187) reduced their A_1C_ by 0.43% from baseline to year 2. -Program completers (n = 155) reduced their A_1C_ by 0.46% from baseline to year 2.
Shane-McWhorter^35^		Interactive voice response	CV health, GC, HBP, MA	Mean A_1C_ decreased from 9.73% at baseline to 7.81% at the end of the program (*P* < .0001). SBP also declined significantly, from 130.7 mm Hg at baseline to 122.9 mm Hg at the end (*P* = .0001). LDL content decreased significantly, from 103.9 mg/dL at baseline to 93.7 mg/dL at the end (*P* = .0263). MA improved, but not significantly.
Skolarus^38^	**Self-determination theory**	Tailored SMS messages	HBP, MA	There were no between-group differences in the change from preintervention to postintervention SBP or DBP (−3.1; 95% CI, −14.4 to 8.3; *P* = .60). The within-IG change in SBP was −11.3 mm Hg (SD, 22.9 mm Hg), and within the CG, it was −14.4 mm Hg (SD, 26.4 mm Hg). Similarly, the within-IG change in DBP was −8.6 (SD, 15.9) mm Hg, and within the CG, it was −9.5 mm Hg (SD, 12.9 mm Hg); this between-group difference was not significant (−0.9; 95% CI, −7.7 to 5.9; *P* = .79). Within the IG, there was no change in MA (*P* = .69). Focus groups: Tailored SMS received unanimous positive responses. Participants reported using their texts to keep a record of their BPs to take to their primary care providers. Overwhelmingly, participants did not want text messages supplemented with phone calls, workshops, cooking demonstrations, or Internet modules. Participants did not want religious content included in their SMS.
Staffileno^39^	**Social cognitive theory**	Web-based education, pedometer	HBP, WL, healthy eating, PA	SBP, DBP, weight, and BMI did not differ across treatment groups. However, on average, there was a −1.2- and −5.6-lb weight loss in the DASH and PA groups, respectively. There was a 0.18 and 0.84 within-group effect sizes for weight in the DASH and PA groups, respectively. Among DASH participants, total DASH scores improved from 1.5 ± 0.5 to 2.9 ± 1.1 (*P* = .001). The largest effects noted were associated with increases in vegetables (0.84), nonfat dairy (0.71), and fruits (0.62), which contributed to a very large total DASH score effect (1.68). With regard to PA participants, the change in daily average steps was trending toward significance (*P* = .055) and corresponded to a favorable (+39%) change in daily steps.
Svetkey^40^	**Social cognitive theory and transtheoretical model of change**	Mobile phone application	WL	The IG lost significantly more weight than controls at 6 mo (net effect, −1.92 kg [95% CI, −3.17 to −0.67]; *P* = .003), but not at 12 and 24 mo.

Abbreviations: BMI, body mass index; BP, blood pressure; CG, control group; CHF, coronary heart failure; CV, cardiovascular; CVD, cardiovascular disease; DASH, Dietary Approaches to Stop Hypertension; DBP, diastolic blood pressure; DMO, digital medicine offering; EHL, eHealth literacy; FRS, Framingham risk score; GC, glycemic control; HbA1c, hemoglobin A_1c_ (glycated hemoglobin); HBP, high blood pressure; HC, hip circumference; HL, health literacy; IG, intervention group; LDL, low-density lipoprotein; MA, medication adherence; mHealth, mobile health; MMAS, Morisky Medication Adherence Score; PA, physical activity; PDA, Personal digital assistant; peak VO_2_, peak oxygen uptake; RHM, Remote Health Monitoring system; SBP, systolic blood pressure; TG, telephone group; UC, usual care; WC, waist circumference; WL, weight loss.

^a^Used as part of the questionnaire, not theory-informed intervention.

#### Mobile Health Features

Mobile health features entailed communication mechanisms, decision support, activity monitoring, and motivation techniques. Most studies were designed to deliver personalized messages that varied in communication mode: automated text messages,^13,14,21,27,32,37^ tailored text messages,^18,24,29,30,33,38^ and prerecorded audio files/interactive voice response.^21,24–26,35^ Some participants received messages multiple times a day^14,15,21,23,26^ or on a weekly basis.^27,32,38^ The researchers allowed participants to choose the number of messages they would receive per day and time of receipt.^34,35^

Most decision tools were used in studies with tracking devices and accelerometers. Predefined prompts were sent to participants for tracking BP, blood glucose, weight, dietary intake, and physical activity. Outside receiving data entry instruction,^20,21,27,30,35^ decision support was also provided when the data reached a critical value.^27,38^ Overall, some coaching was implemented,^13,18,29,34,39,40^ mostly in the form of support and motivation to encourage patient activation, which is defined as having the knowledge, skills, and confidence for self-managing health.^44^

Another innovative feature was gamification, where interactive self-quizzes and trivia were offered on the different mHealth platforms.^14,16,23^ Other studies included reward-based motivators in their programs, such as goal-setting challenges.^16,25^ Virtual communities, social network sites, and accountability groups were used to provide encouragement and reinforcement, including a computer-assisted social support group,^25^ discussion forums for participants,^16^ and a buddy system component within applications to bolster ongoing social support.^40^

### Usability and Acceptability

#### Perceived Ease of Use

Eight studies identified the different mHealth modalities as easy to use.^15,19,22,29,33,35,38,40^ In 1 study, 81% of participants reported that they “did not mind wearing the patch.”^22^ One study affirmed that less demanding application features with “the simplest interactions” were used the most.^40^ To ensure ease of use, participants recommended resolving technical issues, such as bugs and damaged memory cards, before releasing a system.^37^ They suggested mHealth systems should have short tutorials with access to technical support, while also being “intuitive to use, should someone wish to skip any training.”^37^

#### Perceived Usefulness

Participants from 14 studies expressed that mHealth was useful for their daily self-management practices.^17,18,22,24,25,30–32,34–38,40^ Interviewees from a qualitative study “perceived that technology may be useful in increasing their awareness of eating patterns.”^36^ Developers customized systems to meet the users' needs^31^ of vulnerable populations, such as individuals with mental health needs,^32^ low literacy,^16,29,32^ and low English proficiency.^25,35^ Interventions with instantaneous feedback were also deemed useful,^36^ most notably in studies measuring physical activity.^18,24,32^ In cases where high usefulness was reported, participants remained engaged in the program even after completion.^34^

#### Attitude Toward Use

Researchers used various strategies to increase participants' desire towards use, including regularly adding new content^40^ and personalization features.^37^ Participants endorsed having positive attitudes in studies that offered information in multiple languages, especially with high proportions of ethnic minorities.^25^ One study reported that participants had a positive attitude toward mHealth in relation to self-care but were “very concerned about the privacy of their data.”^19^ Overall, participants from 5 studies endorsed high satisfaction with using mHealth,^19,35^ especially tailored text messages.^29,33,38^

#### Intention to Use Mobile Health

Only 2 studies explored participants' intention to use mHealth.^36,37^ In 1 study, most of the participants surveyed reported that they would use mHealth to prevent or manage chronic diseases if it was of no cost to them (ie, smartphone and app were free).^36^ Meanwhile, participants in a qualitative study expressed interest in using activity trackers to monitor their physical activity, stating that this could help them increase their physical activity.^37^ None of the studies included in this review explored the association between intention to use and the actual adoption of mHealth.

#### Mobile Health Adoption and Engagement

Studies that targeted promoting patient activation and changing lifestyles using motivational strategies had high adherence to mHealth.^13,17,29,31^ Participants who had higher perceived disease risks were more adherent to the treatment protocol,^28^ except for kidney transplant recipients.^30^ One article attributed poor adherence to mHealth with low socioeconomic status and health disparity issues, where participants had competing life priorities: lack of childcare, work schedules, and poor healthcare access.^24^

Some studies used various engagement metrics, such as descriptive and correlation statistics, to monitor mHealth use. Glasgow and colleagues^25^ stated: “We calculated the percent of days for which tracking data were entered on the website for each of the three target behaviors. Time spent on the site for each visit was calculated as follows (excluding page view times exceeding 30 minutes): total time on site per visit = (last page visit time – log-in time) + (last page visit time – log-in time)/(n – 1 total pages visited).” They found a low association between patient characteristics and website use (Spearman *r* < 0.20). Their Latino participants, who had low to moderate health literacy, were as equally engaged (number of visits, time spent on the website) in the program as the other participants. This was attributed to their efforts to make the website more culturally appropriate.^25^

Graphs were able to show participants their progress,^20,24,31,37^ which displayed their target goal versus actual steps taken.^24^ Progress bars were added to computer-assisted programs for subjects to track their progress^25^ or received a weekly report describing the percentage of time pills was missed.^31^ Engagement decreased over time for all randomized controlled trials, especially those with longer duration and follow-up periods.

### Effect of Mobile Health Interventions

Primary study outcomes included glycemic control^14,20–22,24,26,34,35^; weight loss, including change in anthropometrics such as waist-to-hip ratio^13,23,24,32,34,39,40^; physical activity/fitness^18,23,25,29,32,39^; medication adherence^14,25,27,28,31–33,35,38^; overall cardiac risk factors^13,17,23,35^; and hypertension control.^5,22–24,27,28,30,31,35,38^ Effect estimate statistics were not performed given the clinical and methodological heterogeneity of the data. Glycated hemoglobin (HbA_1c_) and hypertension were the only 2 outcomes that were measured consistently across studies; however, the number of studies was not enough to run a meta-analysis. Intervention impact is reported descriptively and is also summarized in Table 3.

**TABLE 3:** Identified Research Gaps

Elements of Evidence Gaps	Gaps Identified
Intervention	▪ Lack of programs to manage diet
Sample	▪ Lack of mHealth research specifically assessing immigrant populations
Modalities	▪ Lack of studies using less clinician coaching and more focus on patient activation/self-care
Approach	▪ Lack of CBPR approach ▪ Lack of theoretically driven research
Setting	▪ Lack of research in inner city or resource-poor settings
Outcomes	▪ Lack of outcomes related to chronic disease self-management ▪ Lack of studies looking at patient engagement with application ▪ Lack of studies looking at health literacy and digital literacy

Abbreviations: CBPR, community-based participatory research; mHealth, mobile health.

#### Clinical Outcomes

Five of 11 studies had significantly effective interventions that focused on reducing HbA_1c_,^17,21,22,34,35^ with differences ranging from 0.43% to 1.92% at 3 and 6 months in intervention groups. Most of the studies had an unclear risk of bias,^17,21,34,35^ with the exception of 1 study^22^ with a low risk of bias. Only 1 study reported whether participants were taking oral antihyperglycemics (eg, metformin) versus insulin injections.^22^ Although Forjuoh and colleagues^20^ found no marked reductions in HbA_1c_ for minority persons, there was a reduction in HbA_1c_ for all racial/ethnic groups from baseline to a 2-year follow-up. Similarly, Arora and colleagues'^14^ text-based program did not render a significant reduction in HbA_1c_; however, their results revealed less emergency department utilization among their Spanish-speaking subgroups.

Of the 9 studies measuring hypertension as an outcome, 4 studies reported no change in systolic and diastolic BP across treatment groups.^22,24,38,39^ For the studies that were successful, reduction ranged from 7.8 mm Hg^35^ to 24.1 mm Hg^27^ for systolic BP and 11.3 mm Hg for diastolic BP.^27^ Some studies reported the percentage of participants achieving their goal as follows: 81% at week 4 and 98% at week 12,^22^ 50%,^27^ and 91%.^30^

Six studies researched outcomes in anthropometric measurements.^23,24,32,34,39,40^ They found between- or within-group differences in weight loss or a decrease in waist/hip circumference. Weight loss ranged from 0.81 kg (≅1.78 lb)^32^ to 6.2 kg (≅13.67 lb).^23^ Mobile health modalities for these studies were smartphone applications^23,24,34,40^ and wearable technologies such as a pedometer^39^ and Fitbit.^32^ The greatest change was noted beyond 6 months; however, 1 study reported no changes at 12 and 24 months compared with 6 months.^40^

#### Behavior/Lifestyle Modification Outcomes

Four of 6 studies reported an increase in physical activity.^23,25,29,39^ Studies using trackers/wearable sensors as part of their interventions found significant increases in steps per day.^23,29^ Two studies that monitored physical activity did not have significant results.^18,32^ On the contrary, web-based programs used to promote self-management of CMRFs were successful. For example, 1 study used a highly reliable and validated self-report questionnaire, the Community Healthy Activities Model Program for Seniors. Its items measure physical activity, and the participants reported an increase in physical activity as compared with baseline. Whereas there was a significant relationship between self-monitoring and improvement in physical activity, there was no correlation between engagement strategies and physical activity (Spearman *r* = 0.14, *P* > .05).^25^

The 2 studies that focused on improving eating habits^23,39^ were very successful. One study had greater reductions in intake of saturated fat and sugar-sweetened beverages,^23^ and the second study reported that total Dietary Approaches to Stop Hypertension scores improved from 1.5 ± 0.5 to 2.9 ± 1.1 (*P* = .001)^39^ between the intervention and control groups. The largest effects were correlated with increases in vegetables (0.84), nonfat dairy (0.71), and fruits (0.62), which led to a large total score effect (1.68). Although Glasgow and colleagues^25^ did not study diet as an outcome, they noted that website use was highly related to dietary measures.

Five of 7 studies measuring medication adherence^25,28,33,35,38^ saw no difference between the intervention group versus the control group. Han et al reported the number of participants taking antihypertensives increased from baseline to 16 weeks (from n = 3 to n = 5). Another study saw an improvement on the mean (SD) Morisky Medication Adherence Scale score by 0.4 (1.5) among the intervention group, whereas the score remained unchanged among the control group (between-group difference, 0.4; 95% confidence interval, 0.1–0.7; *P* = .01).^31^

#### Other Outcomes

For the 2 articles studying health literacy, 1 study reported a high health literacy score (84.8% [39/46] with eHEALS score ≅ 26) and found no differences by sex^16^; the second study described effect sizes for hypertension-related health literacy improvement from 0.1 to 1.7.^27^ Austin and colleagues investigated readmission rates for their patients with congestive heart failure and found a 10% readmission rate compared with the Roper baseline rate of 21% (*P* = .047). Another study saw that a change in peak oxygen uptake after 12 weeks was different between the mHealth group (4.7% ± 13.8%) and the usual care group (−8.5% ± 11.5%, *P* < .05).^18^

## Discussion

To the authors' knowledge, this is the first article to systematically review mHealth interventions promoting self-management of CMRFs and how they impact vulnerable populations. Overall, the 28 mHealth studies reviewed were successful in improving physical activity, managing diet, optimizing HbA_1c_ levels, maintaining hypertension control, and promoting weight loss.

Only 3 articles specifically targeted ethnic minorities,^23,27,39^ but most studies did not report on outcome differences between racial and ethnic groups.^14,15,17,18,23–26,29,34,35,38–40^ African Americans have the highest prevalence for type II diabetes^45,46^ and are often understudied in diabetes research.^47^ Likewise, approximately 17% of Latinos within the United States have type II diabetes, compared with almost 8% of non-Hispanic whites,^48,49^ and diabetes disproportionately affects Latino individuals.^48^ Populations with CMRFs often face barriers to healthcare because of social and structural barriers in the community such as transportation, insurance status, and language barriers.^3,50^ In addition, ethnic minorities have low digital literacy compared with non-Hispanic whites.^51,52^ Although researchers are often limited to self-report measures of digital health literacy (eg, eHEALS),^53^ future studies should also measure operational skills of digital literacy with novel self-report tools, such as the Digital Health Literacy Instrument.^54^ Digital literacy requires both cognitive and operational skills, and this tool measures both. Given the known health disparities in CMRFs that exist between nonnative English speakers and native English speakers,^3^ mHealth interventions targeting racial/ethnic minorities should also be culturally sensitive. For example, 2 study showed that sending culturally tailored motivational text messages in Spanish improved high BP outcomes for Latinos.^27^ Indeed, the interventions available in multiple languages were regarded as highly useful by participants.^27^ The public health of Latinos is especially a concern for the United States, given that the Latino population is the largest minority group and is expected to become the largest ethnic group by 2050.^55^ More efforts should be made in meeting participants where they are in the community. In addition, more research is needed to explore the effect of immigrant status or generational differences on the use of mHealth in CRMF management.

The intervention studies reporting high satisfaction and ease of using mHealth were inclusive of their users in the research process.^19,38^ Community-based participatory research offers a comprehensive approach for building rapport with participants, maintaining trust within communities, and developing culturally sensitive interventions.^56^ End users should be collaborators in the mHealth research process, because they can provide genuine feedback on user experience.^57^ Only 2 studies in this systematic review used such an approach to improve CMRF management.^16,40^ Besides leveraging partnerships with participants, researchers in mHealth should also use qualitative and mixed methods research. A comprehensive review of more than 600 studies using mHealth and text messaging for health interventions identified no studies using qualitative research and only 1 study that used mixed methods.^58^ More research is needed to understand the context of using mHealth to manage CMRFs, such as how patients with CMRFs incorporate mHealth into their lifestyles, when they use mHealth, and how they use and/or adapt mHealth to their unique chronic condition needs.

This review found that only 10 of the 28 articles used a theoretical framework, and some constructs investigated did not have operational definitions. Without a precise definition, relationships among variables cannot be determined or tested, which limits the heuristic property of the study design. Most studies reported results on participants' willingness to use mHealth as evidenced by its ease of use and usability, yet there was limited information on attitude and engagement. Some studies used various definitions for engagement,^25,29^ perhaps because there is no tool available to measure how a user actually interacts with mHealth.^59^ Although it is important to understand mHealth adoption, it would be useful to determine how participants engage with mHealth beyond the novelty phase. Longitudinal studies should monitor engagement over a longer period as compared with the average of 3- to 6-month follow-up noted in these studies. In addition to measurement variability, engagement in mHealth should also be evaluated accordingly by monitoring fidelity. Two studies measured engagement by calculating the percentage of days for which tracking data were entered^25^ and by recording the number of log-in times or data usage.^29^ Engagement has predicted better health outcomes in those who use mHealth versus those who do not.^59^ Future research should involve using the technology acceptance model as a framework to guide future mHealth research by considering each construct when discussing engagement with mHealth. For better dissemination, we would be able to propose key mechanisms by which mHealth interventions can influence and sustain behavior change.

### Limitations

Although this study provides a thorough review of available mHealth research for self-management of CMRFs, there are some limitations of the studies that need to be addressed. We restricted studies to those performed in the United States only to explore underserved populations, racial and ethnic minorities. Because of the article's focus on vulnerable populations, it is possible that the synthesis of this review may not be comprehensive. We were unable to estimate the risk of bias over time because there were only 25 records eligible, which was not enough observations for the trend analysis. Instead, we merged all years below 2014, summarized the data by year, and discussed them descriptively. Moreover, because of clinical and methodological heterogeneity, we did not have enough studies addressing the same outcomes to run meta-analyses. Although there were studies in a larger number addressing hypertension, diabetes, and obesity, because of the vast diversities in terms of study design and sample characteristics, we were not able to run meta-analyses.

### Strengths

Despite these drawbacks, our review included both quantitative and qualitative articles, which enhanced knowledge on barriers and facilitators to self-management of CMRFs using mHealth. This review is also in line with the aims of the National Institutes of Health All of Us program,^60^ by revealing gaps in mHealth research with vulnerable populations, as well as specific factors contributing to the uptake, engagement, or efficacy of mHealth in these populations with CMRFs. A large number of the studies extracted were randomized controlled trials, with a high level of quality. Nevertheless, they included large sample sizes, which demonstrated efficacy. The literature search was very thorough, given that all review team members had previous experience conducting systematic reviews. The search was inclusive as possible, consisting of studies published in indexed journals, as well as those found in additional hand search.

## Conclusion

Despite burgeoning mHealth research, this systematic literature review supported that there have been limited mHealth interventions applied to underserved groups. Mobile health presents a promising avenue for eliminating cardiovascular disease health disparities.^19^ The results of this review suggest the need to develop more patient-facing mHealth approaches such as community-based participatory approach, patient-centered research, qualitative inquiry, and mixed methods research. The findings of this review also demonstrate that more theoretically supported mHealth research is warranted. This could serve to not only increase our understanding of how to manage CMRFs but also improve outcomes in health promotion research through mHealth.

**What’s New and Important**

- Research supports that mHealth self-management interventions targeting CMRFs may be effective in increasing physical activity, decreasing weight and waist/hip circumference, and improving diet; however, there were mixed results regarding their effects on medication adherence, BP, and HbA_1C_.
- Although studies include racial and ethnic minorities in their sample, few studies investigate racial/ethnic differences in study outcomes or culturally tailor the mHealth intervention.
- Future research of mHealth self-management interventions should explore racial/ethnic differences in study outcomes, recruit immigrants and patients in resource-poor settings, contextualize mHealth adoption, have a theory-guided intervention, use a community-based participatory research approach to culturally tailor mHealth interventions, and include self-management outcomes.
